# Illuminating the Challenges and Diagnostic Utility of Plasma Microbial Cell-Free DNA Sequencing in Suspected Infective Endocarditis: A Retrospective Observational Cohort Study

**DOI:** 10.1093/ofid/ofaf099

**Published:** 2025-02-17

**Authors:** Myeongji Kim, Pansachee Damronglerd, Sofia Molina Garcia, Zachary A Yetmar, Samrah Razi, Nischal Ranganath, Maryam Mahmood, Omar M Abu Saleh

**Affiliations:** Division of Public Health, Infectious Diseases, and Occupational Medicine, Mayo Clinic, Rochester, Minnesota, USA; Division of Infectious Diseases, Faculty of Medicine, Thammasat University, Pathum Thani, Thailand; Division of Public Health, Infectious Diseases, and Occupational Medicine, Mayo Clinic, Rochester, Minnesota, USA; Department of Infectious Disease, Cleveland Clinic, Cleveland, Ohio, USA; Department of Internal Medicine, Washington University, St. Louis, Missouri, USA; Division of Public Health, Infectious Diseases, and Occupational Medicine, Mayo Clinic, Rochester, Minnesota, USA; Division of Public Health, Infectious Diseases, and Occupational Medicine, Mayo Clinic, Rochester, Minnesota, USA; Division of Public Health, Infectious Diseases, and Occupational Medicine, Mayo Clinic, Rochester, Minnesota, USA

**Keywords:** infective endocarditis, culture-negative endocarditis, microbial cell-free DNA sequencing, molecular diagnostics, next-generation sequencing, metagenomics

## Abstract

**Background:**

Infective endocarditis (IE) is a life-threatening infection often challenging to diagnose, particularly in culture-negative cases. Plasma microbial cell-free DNA (mcfDNA) sequencing has shown potential for detecting pathogens in IE. However, its clinical utility, diagnostic impact, and limitations remain debated. This study evaluates its use in diagnosing and managing IE in a tertiary care setting.

**Methods:**

This single-center retrospective cohort study included adult patients (≥18 years) who underwent mcfDNA sequencing via the Karius test for suspected IE at Mayo Clinic Rochester between December 2019 and February 2024. Diagnostic classification followed the 2023 Duke–International Society of Cardiovascular Infectious Diseases criteria. Data on demographics, clinical features, routine microbiologic workup, and mcfDNA sequencing results were collected. Statistical analysis was conducted to evaluate diagnostic utility and treatment impact.

**Results:**

Among 141 patients, 66 had a diagnosis of IE, with mcfDNA sequencing identifying pathogens in 60.6% of them, compared with 39.4% with routine workup. mcfDNA sequencing was the sole microbiologic test with positive results in 33.3% of patients, leading to antimicrobial adjustments in 50.0% of that group. Clinically insignificant mcfDNA sequence detection occurred in 28.6% of patients without a diagnosis of IE.

**Conclusions:**

mcfDNA sequencing is a valuable adjunctive tool for diagnosing culture-negative IE and guiding antimicrobial therapy when clinical suspicion is high. However, its utility depends on appropriate clinical context, highlighting the need for careful test interpretation and further prospective studies to assess patient-centered outcomes and cost-effectiveness.

## Teaser

Plasma microbial cell-free DNA sequencing has a role in microbiologic diagnosis and antimicrobial management of infective endocarditis if used in appropriate clinical scenarios with high pretest probability.

Infective endocarditis (IE) is a life-threatening infection, with a 1-year mortality rate of 30% [1, 2]. The incidence of IE is rising with advancing life expectancy and the increasing incidence of predisposing comorbid conditions, along with more cases associated with cardiac implantable electronic devices and transcatheter valve replacement [3–5]. Culture-negative IEs are estimated to account for 10%–20% of all IEs, and despite advances in diagnostic assays such as serologic and molecular testing, there remain challenging cases wherein microbiologic diagnosis is elusive [6].

The commercially available Karius test, developed by Karius, detects plasma microbial cell-free DNA (mcfDNA) by next-generation sequencing (NGS). Compared with commercially available multiplex polymerase chain reaction (PCR) panel tests in blood that can detect up to 100 pathogens, depending on the assay [7, 8], mcfDNA sequencing provides the advantage of unbiased detection of more than a thousand DNA pathogens, including bacteria, fungi, virus, and parasites. In contrast to metagenomic PCR/NGS performed on cardiac tissue [9], mcfDNA sequencing of blood allows noninvasive and early testing that may guide IE diagnosis and treatment.

The utility of mcfDNA sequencing in IE diagnosis was first investigated by Shah et al [10] in a prospective study involving 30 patients. The study highlighted 3 promising features of mcfDNA sequencing in IE: concordance with blood cultures, detection of pathogens in culture-negative IE, and diagnostic sensitivity after antibiotic exposure [10]. Eichenberger et al [11] again demonstrated that a causative pathogen in IE remained detectable longer with mcfDNA sequencing than in blood cultures in patients who had received prior antibiotic therapy. A case series of 14 pediatric patients suggested that mcfDNA sequencing can identify a causative pathogen in IE with negative conventional workup [12]. Li et al [13] conducted a prospective study in China of 50 patients who had definite IE according to 2020 modified Duke criteria and applied metagenomic NGS in plasma; among 18 patients with culture-negative IE, 10 had their antibiotic regimen adjusted after the metagenomic NGS result [14]. Another mcfDNA sequencing assay developed by Hugobiotech can detect both DNA and RNA pathogens using reverse-transcriptase and has shown promising diagnostic performance in blood, cerebrospinal fluid, and respiratory specimens [15]. With this emerging evidence, the 2023 Duke–International Society of Cardiovascular Infectious Diseases (ISCVID) criteria for IE incorporated mcfDNA sequencing in its microbiologic major and minor criteria (microbiologic major criterion A.2.i and microbiologic minor criterion E.2) [16].

The clinical value and best practices for use mcfDNA sequencing in the diagnosis and treatment of IE remain debatable. For this retrospective cohort study, we report the experience with the mcfDNA sequencing in various clinical scenarios of suspected IE at a large tertiary hospital in the United States. We aim to quantify the diagnostic utility of mcfDNA sequencing and its impact on treatment decisions, describe clinical contexts where the test affected antimicrobial treatment, and highlight important limitations of using mcfDNA sequencing in the workup of IE.

## METHODS

### Study Design and Data Collection

In this single-center retrospective cohort study, we identified all adult patients aged ≥18 years who underwent mcfDNA sequencing via Karius test as part of the evaluation for suspected IE at Mayo Clinic in Rochester, Minnesota, between 1 December 2019 and 29 February 2024. As this is a retrospective study, the Karius test was not used for the purpose of this research but rather as a part of diagnostic evaluation per the treating clinician's judgment. All identified patient records were independently reviewed. Data pertaining to demographic information, comorbid conditions, predispositions to IE, signs and symptoms, microbiologic and pathologic test results, surgical findings, imaging studies, test date and results of mcfDNA sequencing, clinical notes, and mortality outcome were collected and stored in a secure REDCap database [17].

### Definitions

Each case was classified as definite, possible, or rejected IE according to the 2023 Duke-ISCVID criteria [16]. “Routine microbiologic workup” refers to any diagnostic testing other than mcfDNA sequencing performed to detect causative pathogen for IE, including blood cultures (bacterial, fungal, mycobacterial), serologic testing, and any microbiologic or histopathologic testing performed on cardiac tissue, such as stains for organism, immunologic stains, cultures, and targeted and 16S ribosomal RNA gene PCR. Routine workup and mcfDNA sequencing results were considered “concordant” if (1) both routine workup and mcfDNA sequencing did not reveal a causative pathogen or (2) organism(s) detected by mcfDNA sequencing results were also detected in routine workup. Otherwise, routine workup and mcfDNA sequencing results were considered “discordant.” Positive mcfDNA sequencing results were considered “clinically significant” if (1) the patient met by 2023 Duke-ISCVID criteria for definite or possible IE, (2) the patient received a course of treatment for the IE diagnosis from the treating physician, or (3) mcfDNA sequencing results were concordant with those of routine workup or detected organisms that are known to cause IE based on a literature search.

The rationale for using mcfDNA sequencing was determined through a review of clinical notes, and these indications were categorized into 5 scenarios: (1) there was definite or possible endocarditis but blood cultures remained negative or were expected to be negative due to prior antibiotic exposure (culture-negative IE workup); (2) cardiac imaging incidentally discovered abnormal findings suggestive of IE (incidental imaging; (3) the patient presented with undefined infectious syndrome and had multiple predisposing risk factors for IE (risk factors); (4) the physician wanted to confirm the result of routine microbiologic workup (supportive workup); or (5) mcfDNA sequencing was ordered to monitor treatment response or to investigate relapse of IE (treatment response/relapse).

The impact of mcfDNA sequencing result on the antimicrobial treatment was categorized as (1) initiation of antimicrobials, (2) escalation of antimicrobials (ie, antimicrobials were added or modified to broaden the spectrum of therapy), (3) de-escalation of antimicrobials (ie, antimicrobials were partially discontinued or modified to target a narrow spectrum), (4) discontinuation of antimicrobials (ie, complete discontinuation of all antimicrobials), (5) duration extension of antimicrobials, or (6) no impact on antimicrobial therapy.

### Statistical Analysis

Variables were summarized using descriptive statistics. Medians were used for nonnormally distributed continuous data. All analysis was done using Stata/IC software, version 14.2.

## RESULTS

### Participants

**Figure 1.**
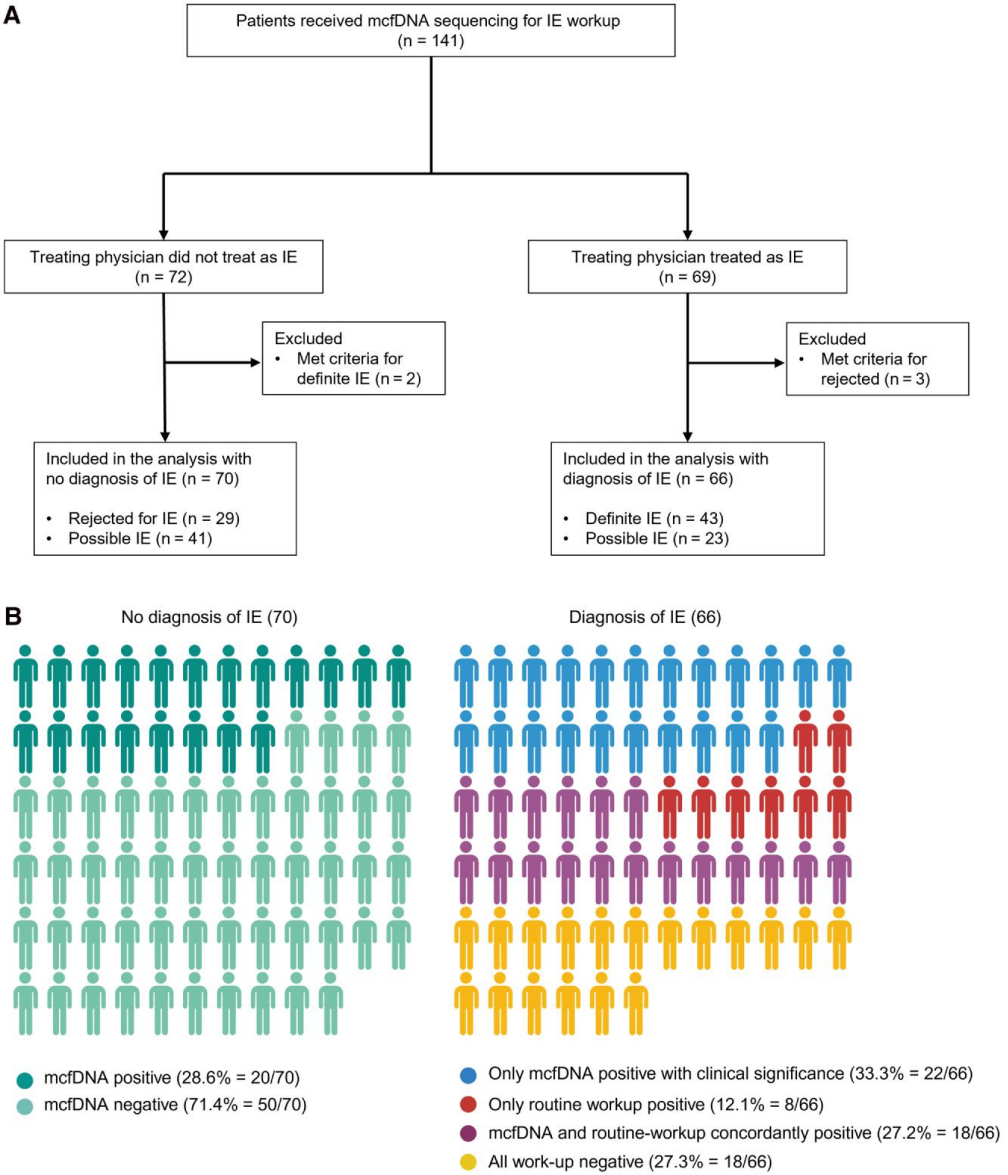
Description of study cohort and results of microbiologic workup. *A,* Flowchart demonstrating diagnostic outcomes in study cohort. *B,* Results of routine microbiologic workup and microbial cell-free DNA (mcfDNA) sequencing in 2 groups of patients with or without diagnosis of infective endocarditis (IE) (created in BioRender by M. K. [2025]; https://BioRender.com/x64w097).

A total of 141 patients were identified. Demographic and clinical characteristics are described in Table 1. The proportion of participants with ≥1 predisposition to IE was high (n = 95 [67.4%]), most notably those with prosthetic valve (n = 66 [46.8%]) and cardiac implantable electronic devices (n = 28 [19.9%]). According to the determination, 70 participants did not have and 66 did have an IE diagnosis, 43 and 23 meeting criteria for definite IE and possible IE, respectively (Figure 1*A*). Five participants were excluded from further analysis due to discrepancies between the treating physician's determination and the diagnosis per 2023 Duke-ISCVID criteria. Among 136 patients included in further analysis, 26 (19.1%) had routine microbiologic workup positive for infectious etiology of IE and 66 (48.5%) had organism(s) detected in mcfDNA sequencing.

**Table 1.** Baseline Patient Characteristics

Characteristic	Patients, No. (%)^a^ (n = 141)
Age (y), median (IQR)	63 (51–72)
Male sex	78 (55.3)
Comorbid condition	
Congestive heart failure	55 (39.0)
Chronic lung disease	29 (20.6)
Chronic kidney disease	38 (27.0)
Liver disease	8 (5.7)
Diabetes mellitus	36 (25.5)
Obesity	35 (24.8)
Immunodeficiency	32 (22.7)
Long-term corticosteroids	9 (6.4)
Biologic therapy	1 (0.7)
Solid organ or bone marrow transplantation	3 (2.1)
Hematologic cancer	4 (2.8)
Solid organ cancer	12 (8.5)
Connective tissue disease	11 (7.8)
Postsplenectomy status	3 (2.1)
Predisposition to IE	95 (67.4)
History of IE	24 (17.0)
Prosthetic valve	66 (46.8)
Previous valve repair	13 (9.2)
Congenital heart disease	21 (14.9)
More than mild regurgitation or stenosis	20 (14.2)
CIED	28 (19.9)
Hypertrophic obstructive cardiomyopathy	0 (0.0)
Injection drug use	6 (4.3)

Abbreviations: CIED, cardiac implantable electronic device; IE, infective endocarditis; IQR, interquartile range.

^a^Data represent no. (%) of patients unless otherwise specified.

### Results of Bacterial Blood Cultures

Among 135 patients who were included in the analysis and had bacterial blood cultures, blood cultures were positive in 13 (9.6%). Among 99 patients who did not receive any dose of antibiotics before blood culture was collected, 12 had positive blood cultures. Among 36 who received any antibiotics before blood cultures were collected, only 1 had a positive blood culture, for *Candida albicans*. No patients who had preceding antibiotics had a blood culture positive for a bacterial pathogen.

### Results of mcfDNA Sequencing

The results of mcfDNA sequencing and routine microbiologic workup are graphically represented in Figure 1*B*. Among 70 patients without a diagnosis of IE, mcfDNA sequencing detected organism(s) in 20 ( 28.6%), representing positive mcfDNA sequencing that is not clinically significant. The organisms detected in these cases and the clinical reasoning for considering these clinically insignificant are summarized in Table 2.

**Table 2.** Summary of Cases in Which Organisms Were Detected in Microbial Cell-Free DNA Sequencing Without Diagnosis of Infective Endocarditis

Patient No.	Organisms Detected (MPM)	Clinical Reason For Ruling Out IE
1	*Streptococcus intermedius* (930)	Patient had disseminated streptococcal infection without evidence of IE
2	*S intermedius* (505)	Firm alternative diagnosis of bacterial peritonitis
3	*Staphylococcus epidermidis* (86)	Multiple blood cultures negative without any receipt of antibiotics
4	*S epidermidis* (108) and *Staphylococcus haemolyticus* (90)	Firm alternative diagnosis of antiphospholipid syndrome
5	*Acinetobacter haemolyticus* (134)	Alternative diagnosis of nonbacterial thrombotic endocarditis in the setting of metastatic cancer
6	*A haemolyticus* (71)	Blood cultures negative without receiving any antibiotics, low MPM, and alternative diagnosis of degenerative valvulopathy
7	*Escherichia coli* (885)	Recent treatment for *E coli* cystitis
8	*Klebsiella pneumoniae* (14 200), *Bacteroides ovatus* (654), and *Bacteroides fragilis* (460)	Concomitant ventilator-associated pneumonia, sputum culture positive for *K pneumoniae* complex
9	*Klebsiella michiganensis* (4324)	Absence of any other findings (ie, normal echocardiogram) to suggest IE
10	*Pseudomonas citronellolis* (98)	Firm alternative diagnosis of bacterial pericarditis
11	*E coli* (198) and *Pseudomonas aeruginosa* (135)	Absence of symptoms or any other findings (ie, normal echocardiogram) to suggest IE and blood cultures negative without recent antibiotics
12	*Veillonella dispar* (25) and *Enterococcus saccharolyticus* (22)	Thought to be low-grade GI translocation in the host with prolonged neutropenia
13	*Bacteroides eggerthii* (1200), *Bacteroides uniformis* (419), and *B ovatus* (394), *Bacteroides thetaiotaomicro*n (317), *Clostridium clostridioforme* (142)	Considered GI translocation, alternative diagnosis of nonbacterial thrombotic endocarditis in the setting of metastatic pancreatic cancer
14	*S epidermidis* (65 030), *Scedosporium apiospermum* (319), and herpes simplex virus type 1 (133)	Firm alternative diagnosis of disseminated *Scedosporium* infection after bone marrow transplant
15	*Neisseria flavescens* (2385)*, Rothia mucilaginosa* (1981)*, Neisseria mucosa* (1936)*, Streptococcus mitis* (1632)*, Prevotella melaninogenica* (1059)*, Morococcus cerebrosus* (809)*, Streptococcus oralis* (756)*, Streptococcus infantis* (574)*, Streptococcus parasanguinis* (409)*, Aggregatibacter segnis* (258)*, Haemophilus parainfluenzae* (251)*, Veillonella parvula* (227)*, Campylobacter concisus* (130)*, Capnocytophaga gingivalis* (124), EBV (95), and *Limosilactobacillus fermentum* (95)	Considered GI translocation, alternative diagnosis was papillary fibroelastoma
16	*Borrelia burgdorferi* (100)	Patient had Lyme disease without evidence of IE
17	EBV (72)	Irrelevant to IE
18	EBV (37)	Irrelevant to IE
19	Human herpesvirus 6B (1412)	Irrelevant to IE
20	*Helicobacter pylori* (226) and EBV (181)	Irrelevant to IE

Abbreviations: EBV, Epstein-Barr virus; GI, gastrointestinal; IE, infective endocarditis; MPM, molecules per microliter.

Among 66 patients with a diagnosis of IE, mcfDNA sequencing revealed a causative organism in 40 (60.6%), and findings of routine microbiologic workup were positive in 26 (39.4%); 18 patients had concordant findings, with both mcfDNA sequencing and routine workup positive for the same organism. Notably, for 22 patients (33.3%), only mcfDNA sequencing was positive. In 8, routine workup revealing infectious etiology for IE, with negative mcfDNA sequencing, as summarized in Table 3. In 18 patients (27.3%) who had a diagnosis of IE and were treated as such, findings of both mcfDNA sequencing and routine workup were negative; among these, 5 qualified for definite IE and 13 for possible IE.

**Table 3.** Summary of Cases in Which Infective Endocarditis Was Diagnosed Clinically and Routine Microbiologic Workup Revealed a Causative Pathogen but Microbial Cell-Free DNA Sequencing Did Not

Patient No.	Findings of Routine Microbiologic Workup	Notes
21	*Cutibacterium acnes* grew in bacterial culture of explanted aortic valve	*C acnes* is not included in the Karius test’s reportable pathogen list
22	*C acnes* grew in bacterial culture of explanted aortic conduit graft	*C acnes* is not included in the Karius test’s reportable pathogen list
23	*C acnes* and *Micrococcus luteus* grew in bacterial culture of explanted tricuspid valve Cor-Knot securing clip	*C acnes* is not included in the Karius test’s reportable pathogen list
24	*Kytococcus schroeteri* grew in tissue from resected mitral annulus, and 16S rRNA PCR detected *K schroeteri*/*Kytococcus aerolatus* DNA	*K schroeteri* is not included in the Karius test’s reportable pathogen list
25	Pathologic examination of excised bioprosthetic aortic valve with Gram staining revealed small foci of bacterial cocci	Findings of blood cultures, valve tissue culture, and 16S rRNA PCR of the valve were all negative, along with mcfDNA sequencing; the patient received antibiotics for 7 d before surgery and for 5 d before mcfDNA sequencing
26	Pathologic examination of excised native aortic valve revealed abundant bacterial cocci	Findings of blood cultures, valve tissue culture, and 16S rRNA PCR of the valve were all negative, along with mcfDNA sequencing; the patient received antibiotics for 8 d before surgery and for 5 d before mcfDNA sequencing
27	Pathologic examination of excised bioprosthetic aortic valve revealed Gram-positive cocci, and initial blood cultures showed *Staphylococcus epidermidis* in 1 out of 2 sets	Valve tissue cultures were negative; the patient received antibiotics for 21 d before surgery and for 16 d before mcfDNA sequencing
28	16S rRNA PCR on debrided aortic annulus detected *Streptococcus sanguinis* DNA	Valve tissue cultures were negative, and pathologic examination did not visualize organisms; the patient received antibiotics for 6 wk before surgery and mcfDNA sequencing

Abbreviations: mcfDNA, microbial cell-free DNA; PCR, polymerase chain reaction; rRNA, ribosomal RNA.

The sensitivity and specificity analysis of mcfDNA sequencing in this retrospective cohort, using Duke-ISCVID criteria as the reference standard, revealed a sensitivity of 60.6%, a specificity of 71.4%, and positive and negative predictive values of 66.7% and 65.8%, respectively.

### Impact of mcfDNA Sequencing on Diagnostic Classification and Treatment Decisions

Among 136 patients, 11 had their diagnostic classification escalated because of the mcfDNA sequencing result; 8 were reclassified from possible to definite IE, and 3 from rejected to possible IE. A total of 20 patients (14.7%) had a change in antimicrobial therapy due to the mcfDNA sequencing result, with de-escalation in 6 (4.4%), initiation in 5 (3.7%), escalation in 4 (2.9%), extended duration in 3 (2.2%), and discontinuation in 2 (1.5%). Among 66 with a diagnosis of IE, 17 (25.8%) had antimicrobial therapy adjusted due to mcfDNA sequencing result, compared with 3 of 70 (4.3%) with no IE diagnosis.

**Figure 2.**
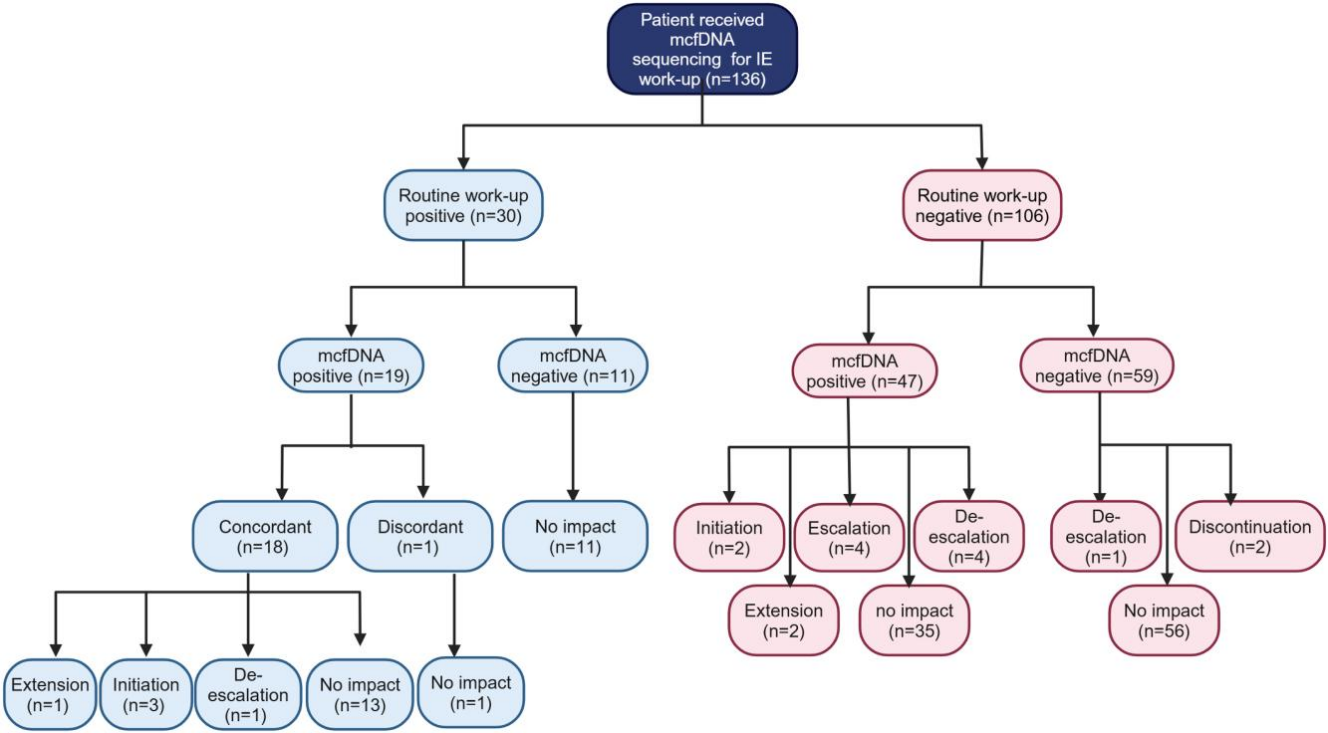
Breakdown of diagnostic scenarios and treatment impact of microbial cell-free DNA (mcfDNA) sequencing result (created in BioRender by M. K. [2025]; https://BioRender.com/y26z221). Abbreviation: IE, infective endocarditis.

Figure 2 demonstrates clinical scenarios of different diagnostic test results and their impact on antimicrobial management. Table 4 summarizes cases in which a mcfDNA sequencing result that was concordant with routine workup altered the antimicrobial treatment. In 3 cases (patients 29, 30, and 33), early antimicrobial therapy optimization was observed as mcfDNA had positive results before routine workup. In patient 31, mcfDNA sequencing increased diagnostic certainty. In patient 32, blood cultures were negative for *Enterococcus faecalis* due to antibiotic exposure and mcfDNA sequencing was more sensitive, leading to more targeted antibiotic therapy. This was later confirmed with a positive culture and 16S ribosomal RNA PCR of the explanted aortic valve.

**Table 4.** Summary of Clinical Cases in Which Microbial Cell-Free DNA Sequencing Concordant With Routine Microbiologic Workup Altered Antimicrobial Therapy

Patient No.	Routine Workup	Organism(s) Detected By mcfDNA Sequencing (MPM)	Diagnostic Class	Treatment Decision	Clinical Reason
29	Q fever serology and PCR positive	*Coxiella burnetii* (1732)	Definite, unchanged	Initiation	mcfDNA sequencing resulted before Q fever serology and PCR resulted
30	*Mycobacterium avium* complex detected in mycobacterial blood culture, aortic tissue, and valve cultures, and 16S rRNA PCR on aortic tissue and aortic valve	*Mycobacterium chimaera* (610) and Epstein-Barr virus (114)	Definite, unchanged	Initiation	mcfDNA sequencing resulted before mycobacterial blood culture resulted and before decision for heart surgery was made
31	*Aerococcus urinae* in blood culture, detected by 16S rRNA PCR of lumbar spine biopsy specimen	*A urinae* (17 699)	Possible, unchanged	Initiation	Blood culture result considered as contaminant; positive mcfDNA sequencing prompted initiation of antibiotics
32	*Enterococcus faecalis* in aortic valve culture and 16S rRNA PCR of aortic valve	*E faecalis* (4738)	Definite, unchanged	De-escalation	Blood cultures were negative, and mcfDNA sequencing led to de-escalation of broad empiric antibiotics to *E faecalis*–directed therapy
33	*M chimaera* in mycobacterial blood culture	*M chimaera* (2295)	Definite, unchanged	Extension	Clinical suspicion confirmed with mcfDNA sequencing before mycobacterial blood culture resulted, leading to extension of empiric mycobacterial therapy

Abbreviations: mcfDNA, microbial cell-free DNA; MPM, molecules per microliter; PCR, polymerase chain reaction; rRNA, ribosomal RNA.

Table 5 summarizes cases in which positive mcfDNA sequencing in the setting of negative routine workup altered the antimicrobial treatment. In these scenarios, mcfDNA sequencing was the only diagnostic test that provided information on a potential causative pathogen, leading to adjustment in antimicrobial therapy to target the identified organism for an appropriate treatment duration. In the single case in which mcfDNA sequencing was discordantly positive, 16S ribosomal RNA PCR of the excised prosthetic aortic valve detected *Streptococcus sanguinis* but mcfDNA sequencing detected only *Helicobacter pylori*, not affecting the treatment plan.

**Table 5.** Summary of Clinical Cases in Which mcfDNA Sequencing Altered Antimicrobial Therapy When Results of Routine Microbiologic Workup Were Negative

Patient No.	Organism(s) Detected With mcfDNA Sequencing (MPM)	Diagnostic Class	Treatment Decision	Clinical Reason
34	*Morganella morganii* (136)	Definite, unchanged	Initiation	mcfDNA sequencing result prompted initiation of treatment for possible *M morganii* endocarditis before surgery was performed
35	*Streptococcus thermophilus* (178)	Changed from possible to definite	Initiation	Positive mcfDNA sequencing result led to diagnosis and treatment for streptococcal endocarditis
36	*Lacticaseibacillus rhamnosus* (166)	Definite, unchanged	Escalation	mcfDNA sequencing result prompted addition of ampicillin to the empiric antibiotics
37	*Bacteroides uniformis* (100)	Definite, unchanged	Escalation	mcfDNA sequencing result prompted addition of anaerobic coverage
38	*Escherichia coli* (298)	Possible, unchanged	Escalation	mcfDNA sequencing result prompted change in gram-negative coverage from ceftriaxone to ertapenem
39	*Acinetobacter haemolyticus* (368)	Possible, unchanged	Escalation	mcfDNA sequencing result prompted escalation of antibiotics from ceftriaxone to cefepime
40	*Corynebacterium amycolatum* (4892) and Epstein-Barr virus (99)	Definite, unchanged	De-escalation	Detection of *Corynebacterium* led to de-escalation of broad empiric antibiotics to *Corynebacterium*-targeted therapy
13	*Haemophilus influenzae* (195)	Changed from possible to definite	De-escalation	mcfDNA sequencing result prompted diagnosis of *Haemophilus* endocarditis, leading to de-escalation of broad antibiotics to *Haemophilu*s-directed therapy
41	*E coli* (193)	Possible, unchanged	De-escalation	mcfDNA sequencing result prompted de-escalation of empiric ciprofloxacin to *E coli*–targeted ertapenem
42	*Streptococcus agalactiae* (17 699)	Possible, unchanged	De-escalation	Broad empiric antibiotics were de-escalated to *S agalactiae*–targeted therapy
43	*Streptococcus intermedius* (691)	Possible, unchanged	Extension	mcfDNA sequencing result prompted diagnosis of possible streptococcal endocarditis, leading to decision to extend ceftriaxone for a total of 6 wk
44	*Staphylococcus haemolyticus* (114 434), *Enterococcus faecium* (29 935), *Enterobacter cloacae* complex (12 513), and *Pseudomonas aeruginosa* (8111)	Change from rejected to possible	Extension	mcfDNA sequencing result prompted treatment of *E faecium* for 6 wk, given the organism's predilection for IE

Abbreviations: IE, infective endocarditis; mcfDNA, microbial cell-free DNA; MPM, molecules per microliter.

### Indications of Ordering mcfDNA Sequencing

**Figure 3.**
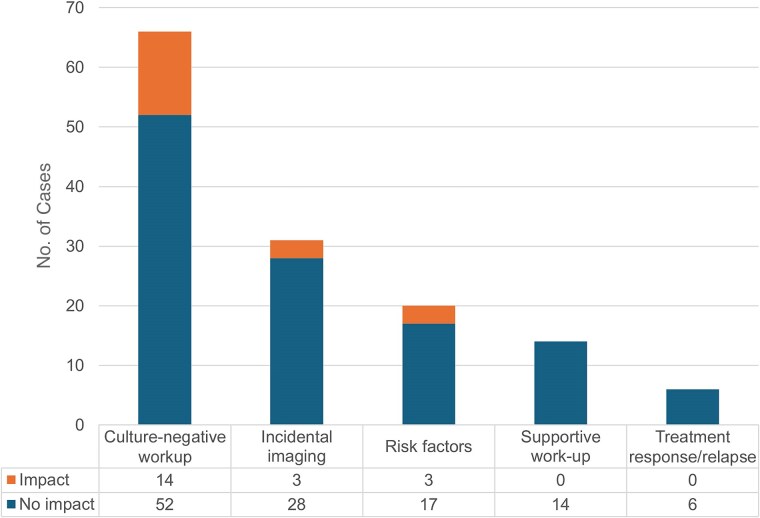
Indications for ordering microbial cell-free DNA sequencing and the numbers of cases in which its results had an impact on antimicrobial therapy.

As summarized in Figure 3, mcfDNA sequencing was ordered in 66 of 136 patients (48.5%) because their clinical presentation was consistent with IE but cultures were negative or expected to be negative due to prior antibiotic administration. In this scenario, mcfDNA sequencing had the highest treatment impact, leading to alteration in antimicrobial therapy in 14 cases (21.2%). It was performed in 31 patients (22.8%) due to incidental findings on cardiac imaging, with treatment impact in 3 (9.7%). It was performed in 21 patients (15.4%) due to extensive predisposition and the presence of risk factors for IE, and antimicrobial therapy was adjusted in 3 (14.3%). When mcfDNA sequencing was ordered to support the result of routine microbiologic workup (14 participants [10.3%]) or to monitor treatment response or relapse (6 participants [4.4%]), it did not have any impact on antimicrobial therapy.

### Association Between Inflammatory Markers and Clinically Significant mcfDNA Sequencing

Among 136 participants, C-reactive protein (CRP) levels were measured in 118 and erythrocyte sedimentation rates (ESRs) in 96 around the time of diagnostic investigation; both CRP and ESR were measured in 94. Among these participants, 22 had negative CRP and ESR findings, applying Mayo Clinic Laboratory's cutoffs of ≤8.0 mg/L for CRP and ≤20 mm/h for ESR. Only 1 of the 22 had clinically significant positive mcfDNA sequencing. Of 26 participants with both positive CRP and ESR findings, 21 had clinically significant positive mcfDNA sequencing.

## DISCUSSION

Despite the updated 2023 Duke-ISCVID criteria and advances in diagnostic technology, diagnosing IE and finding a microbiologic culprit remains challenging. In this study, we highlight the role of mcfDNA sequencing in establishing the microbiologic diagnosis and optimizing antimicrobial treatment of IE. We found that mcfDNA sequencing identified pathogens in 60.6% of cases with a diagnosis of IE, compared to 39.4% with routine workup. Among the remarkable findings, in one-third of the patients (22 of 66) with a clinical diagnosis of IE mcfDNA was the only microbiologic test with positive results, leading to adjustment of antimicrobial treatment in 11 of the 22 (50.0%). However, these numbers should be interpreted with caution as systematic bias could be introduced by a treating physician’s interpreting a positive mcfDNA sequencing result as true-positive when there is definite or possible IE and no other microbiologic workup revealed the pathogen.

Indeed, we found a significant rate of organism detection by mcfDNA sequencing that was not clinically significant. Among 70 patients without a clinical diagnosis of IE, mcfDNA sequencing detected ≥1 microbiologic agent in 20 (28.6%). After exclusion of 4 cases in which clearly irrelevant pathogens were detected (ie, Epstein-Barr virus, human herpesvirus 6B, and *H pylori*), 16 cases remained in which the results of mcfDNA sequencing were subject to the physician's interpretation. The mcfDNA sequencing result was deemed clinically insignificant in the following circumstances: (1) another infectious syndrome is clearly present (Table 2; patients 1, 2, 7, 8, 10, 14, and 16); (2) a firm alternative diagnosis is present (patients 4, 5, 13, and 15); (3) the pretest probability is low (patients 3, 9, and 11); or (4) mcfDNA sequencing detected organisms at a low number of molecules per microliter or detected multiple anaerobic organisms more suggestive of gastrointestinal translocation (patients 13 and 15). These cases highlight the fact that clinical nuance significantly affects the interpretation of the mcfDNA sequencing result and stress the importance of using mcfDNA sequencing only when the clinical scenario is appropriate.

There were fewer cases of false-negative mcfDNA sequencing in which routine workup showed microbiologic etiology (Table 3). There are plausible explanations for negative mcfDNA sequencing results in all 8 cases: in patients 21–23 the organism was not part of the reportable pathogen list for the Karius test, and patients 25–28 received ≥5 days of antibiotics before sequencing. Regarding the first possibility, Karius does not report *Cutibacterium acnes* as a pathogen even if the DNA is detected, due to difficulty with interpreting its clinical significance. Of note, all 8 of these cases had microorganisms detected in the explanted cardiac tissue through histopathologic examination, PCR, or culture, emphasizing that direct examination of surgical tissue is still of paramount importance.

Interestingly, despite extensive evaluation, we noted a “workup-negative” IE cohort, comprising 18 patients who did receive a diagnosis of definite or possible IE but in whom results of all microbiologic investigations were negative, including mcfDNA sequencing. Of these 18 patients, 5 met definite IE criteria and 13 met possible IE criteria per the 2023 Duke-ISCVID guidelines. This group highlights the challenges of establishing a microbiologic diagnosis of IE and the importance of a multimodal approach. The challenge of reaching diagnostic confidence is also highlighted in the observed discrepancy between diagnostic classification according to 2023 Duke-ISCVID criteria and according to the clinician's judgment; 2 patients who met criteria for definite IE were not treated for IE, and 3 who were rejected for IE were treated for IE in our study cohort.

Among 22 participants who had normal CRP and ESR findings, only 1 had positive mcfDNA sequencing that was clinically significant. We hypothesized that the degree of systemic inflammation reflects the ongoing bacterial burden and the infectious process, correlating with a higher amount of plasma mcfDNA.

The current study has several limitations worth noting. In cases with low diagnostic certainty of IE, there was increased subjectivity in the interpretation of mcfDNA sequencing results. When clinical suspicion for IE was lower, the mcfDNA sequencing was more likely to be interpreted as negative. Conversely, when mcfDNA sequencing detected an organism in a case of uncertain diagnosis, physician tended to interpret mcfDNA as positive and treat the case as IE. There is also selection bias in the patient population, as this was a single-center study at a large tertiary referral hospital. In addition, this was a retrospective study performed only in the patients who received mcfDNA sequencing as part of the evaluation of IE, not in all patients with suspected IE. This bias is represented by the analysis showing that our cohort had a high rate of predisposing factors for IE and a low blood culture positivity rate. Given our study’s retrospective nature, we could not calculate the true sensitivity or specificity of mcfDNA sequencing generalizable to all patients presenting with IE.

Finally, the approach for ordering mcfDNA sequencing proposed below might not be applicable in a resource-limited setting or outside the United States, where mcfDNA sequencing is not as readily available. While the Karius test entails significant cost (approximately $2000), early detection of pathogens through mcfDNA sequencing may help reduce the cost of extensive conventional culture-negative IE tests.

**Figure 4.**
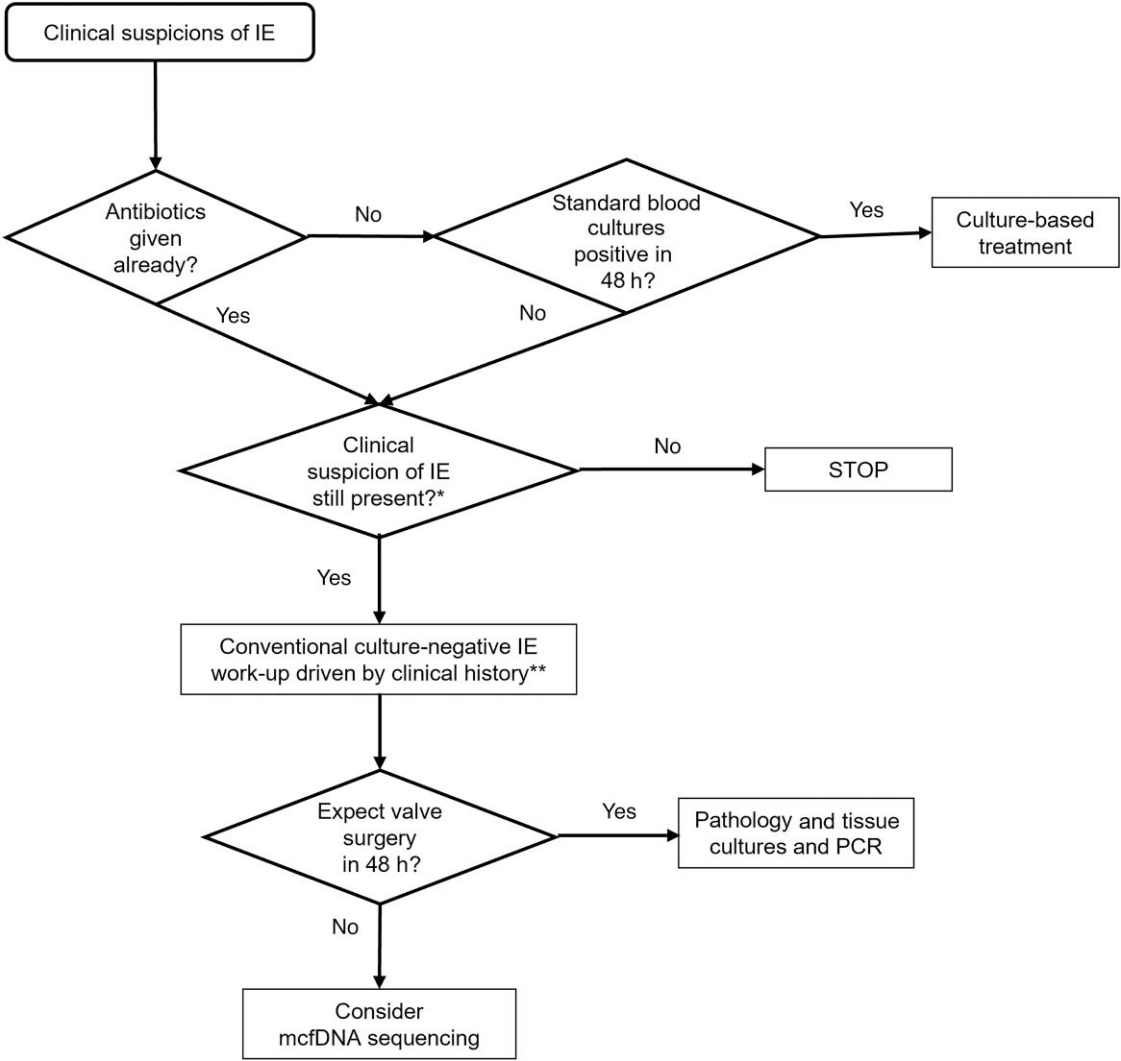
Proposed algorithm for the timing of plasma microbial cell-free DNA (mcfDNA), sequencing in the workup of infective endocarditis (IE). *Consider clinical course, results of imaging studies, and inflammatory markers. **Consider using serologic and molecular diagnostics in accordance with the patient's exposure history and clinical course. Abbreviation: PCR, polymerase chain reaction.

In conclusion, mcfDNA sequencing should be used to help establish the microbiologic etiology of confirmed or anticipated culture-negative IE only when the pretest probability is high (ie, the patient has a syndrome consistent with IE and has signs of systemic inflammation). Specifically, mcfDNA sequencing should not be ordered as a rule-in or rule-out test, nor to diagnose IE in the absence of a consistent clinical syndrome. We propose an algorithm guiding clinicians on the use of mcfDNA sequencing as an adjunctive test to establish a microbiologic diagnosis and guide treatment when there is sufficient evidence of IE (Figure 4).

Randomized, prospective studies assessing the utility of mcfDNA sequencing in all patients with suspected IE would be ideal for determining diagnostic accuracy and the impact on patient outcomes. Patient-centered outcomes, such as mortality rate, length of stay, and antimicrobial adverse reactions, need to be studied. Finally, considering the cost of mcfDNA sequencing, cost-benefit analysis would be valuable.
